# 

**Published:** 1858-09

**Authors:** 


*Nos.* 1 *and* 2 *of Vol. XI, June and July,* 1855.—We will cheerfully credit 50c. to any one who will send us these two numbers.

